# President’s Address

**Published:** 1890-04

**Authors:** J. R. Callahan

**Affiliations:** Cincinnati, O.


### President's Address.

BY J. R. CALLAHAN, D. D. S., CINCINNATI, O.

Read before the Missisippi Valley Dental Association, at Cincinnati, O., March, 1890.

We are now assembled for the 46th annual session of this old and time honored Mississippi Valley Dental Society. As I look back over the records of the past doings of this society, I can see where its founders builded better than they knew. I imagine this society in its earliest days had more to do with bringing dentistry to its present high standing than most of us give her credit for doing. At the very beginning and formation of this society, the founders stepped upon the very highest rounds of professional dignity and honor, and I have no doubt but that they lived up to their theories fully as closely as we of to-day. The preamble to the constitution adopted at the first meeting, Aug. 10, 1844, indicates the elevated and gentlemanly tone of the founders of the society.

In the very beginning they put the seal of their disapproval on charlatanism, amalgam fillings, advertising, derogatory remarks about one another in regard to poor ability, etc.; in fact, it is hard to find anything that the most advanced of us to-day condemn that they did not speak of in no uncertain tone forty-six years ago. Many are the good things done by this society in early days; it has sown seed that has produced an hundred fold; many of the dental societies of to-day are offsprings of this old mother association.

Not the least among the good fruits of this society are the *Dental Register* and Ohio Dental College. I quote from Betty's paper: "So long as our association exists it may point with pride to its record of usefulness in achieving the great feats of having been the progenitor of the oldest existing dental periodical except one in the world, and the second oldest dental college, both of which make their influence felt all over the civilized world."

The society spent money freely, both in the practical and theoretical matters of the profession; they gave prizes for papers to the value of $100; gave medals—gold and silver—for improved appliances. They seemed to be in great earnest in every way, and they did not forget to have good times, too, as they went along. From the records I find that they were wont to gather about the festal board and break bread, crack chestnuts, and have a good social time, and at the close have what they choose to call interlocutory discussion. (By way of parenthesis allow me to say we had an interlocutory discussion a few evenings since, five of us only; we all agreed that it was very profitable and enjoyable). I will read you one or two entries from the record of the meeting held in Louisville, in 1851:

The society, on motion of Dr. Griffith, adjourned to his house to test the utility of their masticating organs in preparing food for the digestive apparatus. The result was proof conclusive that their dental organs, with their muscular attachments, were in good order. This must have been at a 12 o'clock dinner, for they met again at 3 P. M. same day, and at the close of the session adjourned to meet at the residence of Dr. Goddard at 7½ o'clock. The record read as follows: The society met pursuant to adjourment; Dr. Goddard at the head of the table; the presiin the chair, and *every* member present. The discussion on the present occasion was elaborate. Each member defining well his position. All was to the point, all in order and evinced the hospitality of our Kentucky brethern. On the next evening of the same meeting the society met at the residence of Dr. Somerby. The following is the record : Society met at the residence of Dr. Somerby, and the first thing in order was an examination, *vi et dentales*, of a bountiful supper done up in the Doctor's happy manner. The discussion at table elicited much good feeling, which was not interrupted until after an adjournment to the parlor, when some one (who, it is supposed, had been eating too much of the Doctor's candy) introduced the subject of exposed nerves, whereupon the president took the chair and called the house to order and a very *nervous debate* sprang up, discussing the pain of such an exposure. The propriety of re-covering or capping—of killing and filling. Dr. Somerby remarked that he thought it not in accordance with true pathological principles to retain a tooth in the mouth after the nerve had been destroyed, and that the operation of plugging over an exposed nerve by capping or otherwise would generally prove useless, and the idea of repeatedly *tickling* the nerve to make it cover itself with new bone, was more amusing than profitable. This must have been a very enjoyable meeting and the secretary must have been some relation to my friend, Dr. C. M. Wright.

I found in the records many little points of interest, among others that on Sept. 11, 1849, Dr. J. Taft read a paper on the Extraction of Teeth. That must have been among Prof. Taft's first papers ; how many he has written since that time would be interesting to know.

Since we met last year death has claimed some of our members. The grim monster has taken from us some of our very best and most useful men ; those whom we can but ill afford to lose and whose places will long be vacant. Some of them were among the charter members of this society; the others have been among the strong pillars that have made the record of this society what it is. I will leave the names and virtues of these men to the Committee of Necrology.

In the early years of this society it was truly *the* dental society of the Mississippi Valley. It drew its membership from all parts of this great valley and often dentists were in attendance from over the Alleghenies. It was looked upon, and was truly, *the* dental society of the west for many years; but in the forty-six years of its existence a new state of affairs has come about; in almost every State there are Local, District and State Societies and all auxillary, more or less, to the American Dental Society. Under this arrangement the Mississippi Valley Society is left somewhat isolated and has lost much of its former prestige. It has become somewhat local in its management and it is with much difficulty that the programs are filled up each year. Men who write papers say, I have to attend my local society so often I don't see how I can add another society to my already heavy burden, and the workers in dental society affairs are saying quietly, but with much significance, I wonder if the old Mississippi Valley Dental Society has not out-lived her usefulness. As for myself, I will not try to answer the question, it is a serious question and deserves thoughtful attention.

